# Mutant p53 targeting by the low molecular weight compound STIMA‐1

**DOI:** 10.1002/1878-0261.12065

**Published:** 2017-05-02

**Authors:** Nicole Zache, Jeremy M.R. Lambert, Nina Rökaeus, Jinfeng Shen, Pierre Hainaut, Jan Bergman, Klas G. Wiman, Vladimir J.N. Bykov

This article corrects: Mutant p53 targeting by the low molecular weight compound STIMA‐1, Volume 2, Issue 1, 70–80. Article first published on 7 March 2008. https://doi.org/10.1016/j.molonc.2008.02.004

By mistake, the same DAPI control staining picture was shown twice in Fig. 6D. The authors have repeated the experiment, and a revised figure is shown below:

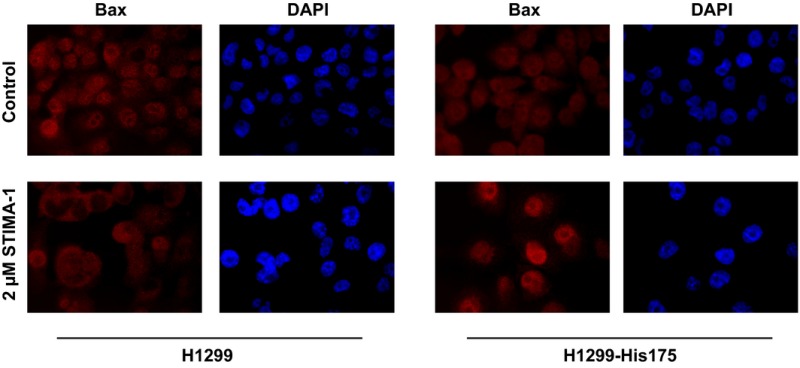

H1299 and H1299‐His175 cells were seeded in six‐well plates with glass slides, 200 000 cells per well. The next day, cells were treated with 2 μm STIMA‐1. After 24‐h treatment, cells were washed twice in PBS, fixed for 10 min at room temperature with 4% paraformaldehyde (PFA), permeabilized for 2 min in 0.2% Triton in PBS and washed 3 x 5 min in PBS. Anti‐Bax antibody SC6236 (Santa Cruz, CA) was diluted 200 times in 2% BSA and incubated with the slides for 2 h at room temperature. After washing 3 × 10 min with PBS, slides were incubated with Alexa Fluor 594 goat anti‐rabbit Ig antibody (Invitrogen/Thermo Fisher Scientific, Sweden), diluted 500 times in 2% BSA, for 1 h, then washed 3 × 10 min in PBS and mounted with Vectashield hard set with DAPI (Vector Laboratories, CA). Images were obtained with a Zeiss Axioplan 2 microscope with an AxioCam HRm Camera.

